# Clinical characteristics, risk factors, and outcome of *Enterococcal bacteraemia*: Assessment of the DENOVA score in a diverse patient cohort

**DOI:** 10.1007/s15010-026-02856-1

**Published:** 2026-06-17

**Authors:** Pia Wehage, Jan Fuge, Luca Hilberink, Ralf-Peter Vonberg, Liz Kinast, Susanne Simon, Oana Joean, Jessica Rademacher

**Affiliations:** 1 Department of Nephrology and Hypertension, Hannover Medical School, Carl-Neuberg-Str. 1, 30625 Hannover, Germany; 2 Department of Respiratory Medicine and Infectious Diseases, Hannover Medical School, Carl-Neuberg-Str. 1, 30625 Hannover, Germany; 3 Biomedical Research in Endstage and Obstructive Lung Disease , Member of the German Centre for Lung Research, Hannover, Germany; 4 Institute for Medical Microbiology and Hospital Epidemiology , Hannover Medical School, Hannover, Germany; 5 Institute of Infectious Diseases and Infection Control , Jena University Hospital, Jena, Germany

**Keywords:** Enterococcus spp., Bacteraemia, DENOVA score, Infective endocarditis

## Abstract

**Background:**

Enterococcal bacteraemia is associated with high mortality rates ranging from 14% to 50% and is one of the most common causes of infective endocarditis. The DENOVA score is a validated screening tool for patients with monomicrobial *E. faecalis* bacteraemia who require echocardiography to rule out infective endocarditis.

**Methods:**

Adult patients hospitalized at Hannover Medical School during the years of 2021 and 2022 with at least one paired blood culture positive for *Enterococcus* species were included in this retrospective observational study. Risk factors associated with in-hospital mortality were identified through a multivariable analysis using logistic regression. Clinical differences associated with the underlying pathogen were analysed. The performance of the DENOVA score for predicting infective endocarditis in enterococcal bacteraemia was evaluated in a high-risk cohort outside its validated application for monomicrobial *E. faecalis* bacteraemia.

**Results:**

A total of 271 cases of enterococcal bacteraemia were included (65.7% *E. faecium*, 31.4% *E. faecalis*, 3% other enterococci). Patients with *E. faecium* bacteraemia had more vancomycin-resistant cases, longer hospital stays and a higher in-hospital mortality rate in comparison to *E. faecalis* bacteraemia. The Charlson Comorbidity Index, the Pitt Bacteraemia Score, and *E. faecium* bacteraemia were all factors independently associated with in-hospital mortality. 5% of enterococcal bacteraemia resulted in infective endocarditis (10.6% *E. faecalis* vs. 3.4% *E. faecium*). Applying the DENOVA score in our cohort let to a sensitivity of 86.7% and a specificity of 67.5% for predicting infective endocarditis.

**Conclusion:**

Enterococcal bacteraemia remains a clinical challenge with high morbidity and mortality. Our results suggest that the application of the DENOVA score might be possible in a high-risk cohort and even beyond its original validation. Future research should focus on personalized risk stratification and tailored management strategies to improve patient outcomes.

**Supplementary Information:**

The online version contains supplementary material available at https://doi.org/10.1007/s15010-026-02856-1.

## Introduction

 Enterococci are Gram-positive commensals in the human intestinal flora, which were historically assigned to the D streptococcus group due to the presence of the D polysaccharide antigen [1]. *E. faecium* and *E. faecalis* are the most commonly encountered species causing bloodstream infections (BSIs). Their intrinsic resistance to cephalosporins and the rising number of resistant strains in the past years - especially ampicillin and vancomycin resistant enterococci (VRE) among *E. faecium* - pose a significant burden on the healthcare system [2–4]. Elderly, immunocompromised patients with multiple comorbidities are at a particularly high risk of enterococcal infection [5–7]. Enterococcal bacteraemia is associated with high mortality rates, ranging from 14% to 50% in recent studies [6, 8, 9]. Inappropriate therapy, vancomycin resistance and missing infectious disease consultation were associated with an increase in mortality [10–12].

Enterococci, particularly *E. faecalis* in over 90% of the cases, are the third most common cause of infective endocarditis (IE) [13]. The NOVA score was developed by Bouza et al. and validated by Dahl et al. to identify patients with enterococcal bacteraemia who require echocardiography to rule out endocarditis [14, 15]. The updated DENOVA score was validated by Berge et al. and showed a superior sensitivity and specificity compared to the NOVA score as a screening tool for monomicrobial *E. faecalis* bacteraemia [16]. A transoesophageal echocardiography is recommended in patients with a DENOVA score of at least three points [16]. The score’s variables include unknown origin of bacteraemia, number of positive blood cultures (BCs) ≥ 2, prior valve disease, auscultation of new heart murmur, duration of symptoms ≥ 7 days, and signs of embolization [16]. The DENOVA score as a screening tool for infective endocarditis was highly sensitive (100%) and had a specificity of up to 85% [16, 17]. However, the score was only validated for monomicrobial *E. faecalis* bacteraemia. Our aim was to test if the application of the DENOVA score could be possible outside the original validated population and extended to *E. faecium* or polymicrobial bacteraemia in a high-risk patient cohort.

The differentiation between bacteraemia caused by *E. faecium* or *E. faecalis* is crucial because it directly influences diagnostic and treatment options as well as prognosis. It is already known that *E. faecium* bacteraemia is more often associated with antibiotic resistance, hospital-acquired infection, higher mortality rates, and an abdominal focus of infection whereas *E. faecalis* bacteraemia is more likely to have a genitourinary origin [6, 7, 18]. The primary aim of this study was to describe the clinical characteristics and outcome of enterococcal bacteraemia in a high-risk cohort and analyse the differences between *E. faecalis* and *E. faecium* bacteraemia.

Hannover Medical School as a university medical centre is characterized by its high number of immunocompromised patients, which is in part due its status as one of Europe’s largest transplant centres. This medically complex, high-risk patient cohort was deliberately selected. Understanding the clinical characteristics of enterococcal bacteraemia and the application of the DENOVA score in such a specialised setting where infections are relevant and challenging enables valuable insight.

## Study design and methods

**Fig. 1:**
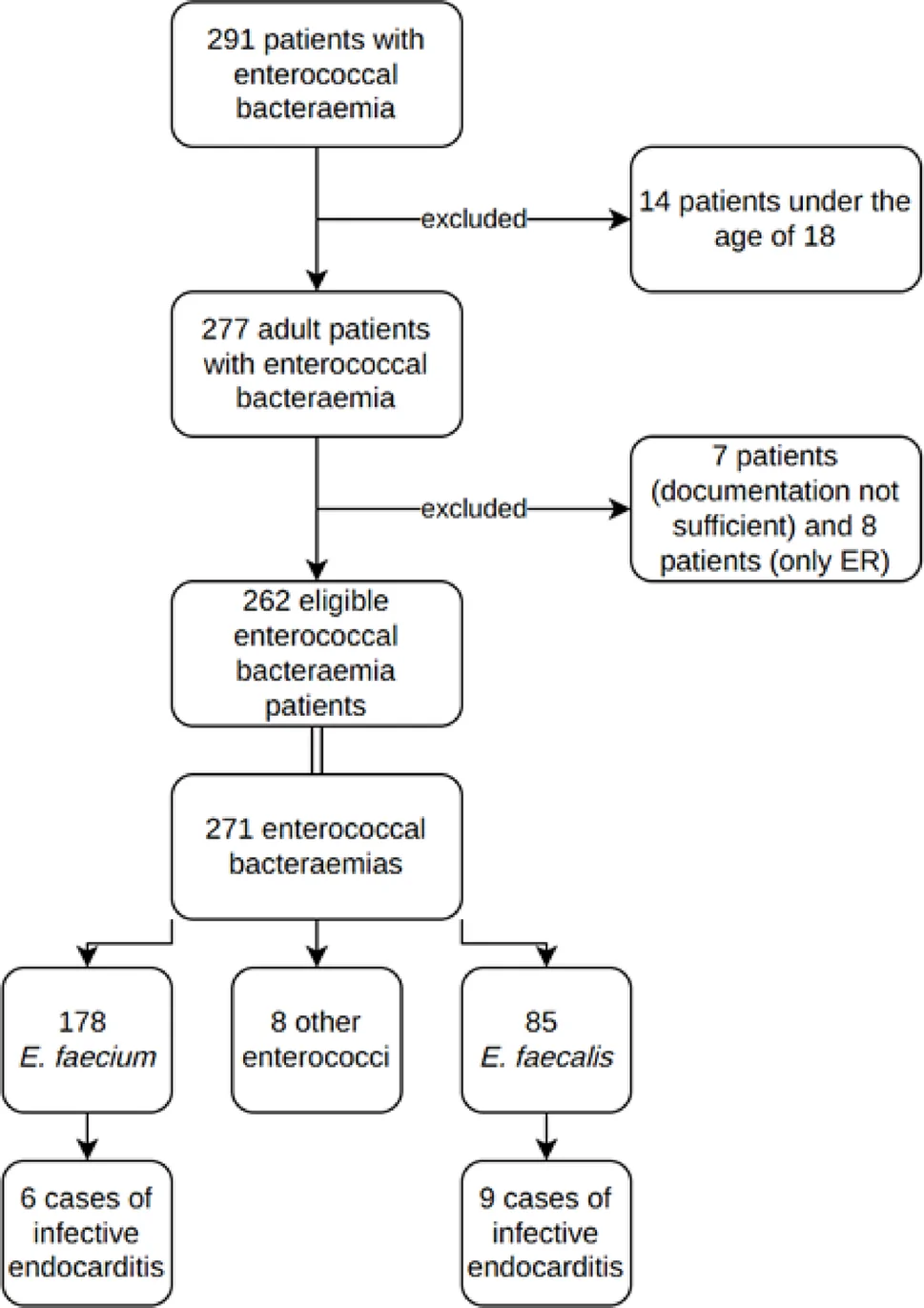
Flow diagram of patients with enterococcal bacteraemia (ER=Emergency Room)

We conducted this retrospective observational study at Hannover Medical School (MHH), a 1520 bed university medical centre in Lower Saxony, Germany. All adult patients hospitalized at Hanover Medical School during the years of 2021 and 2022 with at least one paired blood culture positive for *Enterococcus* species were included. Patients under the age of 18, without sufficient documentation, meaning a doctor’s letter, or patients who were only seen in the emergency room and not admitted to a hospital ward were excluded (Fig. 1). Repeated episodes of enterococcal bacteraemia were only included when they occurred after a clinically relevant time interval and were judged to represent independent infectious episodes. The microbial data to identify suitable patients was obtained from the Institute for Medical Microbiology and Hospital Epidemiology. The study was approved by the Ethics Committee of Hannover Medical School (number 10994_BO_K_2023). Demographics and clinical data were taken from the archive system ALIDA.

Enterococcal bacteraemia was defined as identification of an enterococcal species from one or more paired BCs. The antibiotic susceptibility and the time to positivity (TTP) were tested by the Institute for Medical Microbiology and Hospital Epidemiology at Hannover Medical School. When a second non-enterococcal organism was detected in a paired BC, regardless of its potential clinical relevance, the enterococcal bacteraemia was classified as polymicrobial bacteraemia. VRE screening was considered completed when a rectal swab or a stool culture was tested for VRE during the hospital stay. If the first positive paired BC was drawn 48 h or more after the hospital admission the bacteraemia was considered hospital-acquired [19]. The focus of infection was determined by taking the clinical assessment and diagnostic results from imaging and microbial tests into consideration and was discussed with infectious disease specialists. The maximum Pitt Bacteraemia Score [20] was calculated, and the presence of catheters was assessed on the day of the index BC.

The DENOVA score was calculated based on documented data from electronic health records [16] and IE was diagnosed according to the Duke criteria [21]. All paired BCs, which were positive for *Enterococcus* species during one case of bacteraemia, were added up. A follow-up BC is defined as a BC drawn 24 h or later after the index BC.

Antibiotic treatment was evaluated as adequate when one of the following antibiotics was given either as monotherapy or combination therapy and the isolate showed susceptibility in microbial testing: ampicillin, ampicillin-sulbactam, vancomycin, daptomycin, linezolid, teicoplanin, piperacillin-tazobactam. The therapy was defined as “Combination therapy” when at least two different antibiotics were administered and defined as “Sequential antibiotic therapy” when the method of medication administration was converted from intravenous to oral. The adequacy of the dosing and duration of therapy was assessed independently by two infectious disease specialists. Source control includes all forms of procedures like drainage or debridement that aim at removing the focus of infection.

The patients’ comorbidities were assessed by calculating the Charlson Comorbidity Index (CCI) [22, 23] on the day of hospital admission and their frailty by the Clinical Frailty Scale (CFS) [24]. The patient was classified as “Immunocompromised”, if one of the following criteria was fulfilled: primary immunodeficiency, absolute neutrophil count below 500 cells/µl, chemotherapy, malignancy, solid organ transplant, HIV infection with a CD4 T cell count below 200/µl, bone marrow or stem cell transplant, treatment with systemic steroids at a dose equivalent to at least 20 mg prednisolone per day, immunosuppressive or immunomodulating biological therapy, or disease-modifying antirheumatic drugs (DMARDs).

The length of hospital stay in days was calculated from the date of admission to the date of discharge, regardless of the reason for the discharge, or date of death. The duration of bacteraemia was defined as the number of days between the index BC and the first negative BC if the BC remained negative after an incubation time of seven days. If no follow-up BCs have been drawn, the duration of bacteraemia could not be calculated. Follow-up blood cultures were not drawn at standardized intervals.

**Table 1:** Patients’ characteristics

	Total number, *n*=271 (100%)	*E. faecium*, *n*=178 (65.7%)	*E. faecalis*, *n*=85 (31.4%)	*P* value (*E. faecium* vs. *E. faecalis*)	Other enterococci *n*=8 (3.0%)
Age (years), median (IQR)	62.00 (52-71)	61.00 (50-69)	67.00 (54.5-76.5)	0.001^3^	53.00 (45.75-61.25)
Male Sex	178 (65.7)	111 (62.4)	63 (74.1)	0.059^2^	4 (50.0)
*Comorbidities*					
Solid tumour	55 (20.3)	34 (19.1)	19 (22.4)	0.539^2^	2 (25.0)
Hematologic malignancy	54 (19.9)	47 (26.4)	7 (8.2)	**˂0.001** ^**2**^	0
Cardiovascular disease	163 (60.1)	104 (58.4)	57 (67.1)	0.179^2^	2 (25.0)
Chronic respiratory disease	73 (26.9)	49 (27.5)	24 (28.2)	0.905^2^	0
Chronic kidney disease	108 (39.9)	69 (38.8)	36 (42.4)	0.578^2^	3 (37.5)
Chronic liver disease	81 (29.9)	61 (34.3)	14 (16.5)	0.003^2^	6 (75.0)
Immunosuppression	154 (56.8)	110 (61.8)	40 (47.1)	0.024^2^	4 (50.0)
Diabetes mellitus	80 (29.5)	53 (29.8)	26 (30.6)	0.893^2^	1 (12.5)
Charlson Comorbidity Index (CCI), median (IQR)	5.00 (3-7)	5.00 (3-7)	6.00 (3-8)	0.572^3^	4.50 (2.25-8.75)

**Table 2:** Characteristics of enterococcal bacteraemia

	Total number, *n*=271 (100%)	*E. faecium*, *n*=178 (65.7%)	*E. faecalis*, *n*=85 (31.4%)	*P* value (*E. faecium* vs. *E. faecalis*)	Other enterococci, *n*=8 (3.0%)
Polymicrobial bacteraemia	160 (59.0)	112 (62.9)	42 (49.4)	0.038^2^	6 (75.0)
*Antibiotic resistance*					
Linezolid	5 (1.8)	5 (2.8)	0	0.178^1^	0
Vancomycin	46 (17.0)	45 (25.3)	0	**˂0.001** ^**2**^	1 (12.5)
Ampicillin	177 (65.3)	176 (98.9)	0	**˂0.001** ^**2**^	1 (12.5)
*Mode of acquisition*				**˂0.001** ^**2**^	
Hospital-acquired	205 (75.6)	164 (92.1)	37 (43.5)		4 (50.0)
*Focus of infection*				**˂0.001** ^**1**^	
Unknown	108 (39.9)	80 (44.9)	25 (29.4)		3 (37.5)
Abdominal	78 (28.8)	61 (34.3)	12 (14.1)		5 (62.5)
Genitourinary	42 (15.5)	16 (9.0)	26 (30.6)		0
Catheter-related	22 (8.1)	14 (7.9)	8 (9.4)		0
*VRE screening*				**˂0.001** ^**2**^	
Not carried out	105 (38.7)	54 (30.3)	48 (56.5)		3 (37.5)
Positive	67 (24.7)	61 (34.3)	6 (7.1)		0
Negative	99 (36.5)	63 (35.4)	31 (36.5)		5 (62.5)
Pitt bacteraemia score median, (IQR)	1.00 (0-4)	2.00 (0-5)	1.00 (0-3)	0.310^4^	0 (0-2.25)
*Risk factor*					
Prior use of broad-spectrum antibiotics in the past 6 months	233 (86.0)	170 (95.5)	57 (67.1)	**˂0.001** ^**2**^	6 (75.0)
*Surgery in the past 6 months*					
Abdominal	43 (15.9)	29 (16.3)	12 (14.1)	0.649^2^	2 (25.0)
Urologic	21 (7.7)	10 (5.6)	11 (12.9)	0.040^2^	0
Cardiothoracic	46 (17.0)	38 (21.3)	7 (8.2)	0.008^2^	1 (12.5)
*Intervention in the past 6 months*					
Endoscopy	120 (44.3)	92 (51.7)	22 (25.9)	**˂0.001** ^**2**^	6 (75.0)

DENOVA score	Total number, *n*=271 (100%)	*E. faecium*, *n*=178 (65.7%)	*E. faecalis*, *n*=85 (31.4%)	*P* value (*E. faecium* vs. *E. faecalis*)	Other enterococci, *n*=8 (3.0%)
Origin of infection unknown	108 (39.9)	80 (44.9)	25 (29.4)	0.016^2^	3 (37.5)
DENOVA Score, median (IQR), *n*=270	2.00 (1-3)	2.00 (1-3)	2.00 (1-3)	0.663^4^	1.00 (1-2)
TTE performed after positive blood culture	97 (35.8)	64 (36.0)	31 (36.5)	0.935^2^	2 (25.0)
TEE performed after positive blood culture	33 (12.2)	15 (8.4)	16 (18.8)	0.014^2^	2 (25.0)
Infective endocarditis	15 (5.5)	6 (3.4)	9 (10.6)	0.024^1^	0
*Blood cultures*					
Follow-up blood cultures, *n*=269	194 (72.1)	134 (75.7)	55 (65.5)	0.084^2^	5 (62.5)
Positive follow-up blood cultures, *n*=194	69 (35.6)	50 (37.3)	19 (34.5)	0.720^2^	0

Therapy					
Anti-infective therapy, *n*=267	267 (98.5)	175 (98.3)	84 (98.8)		8 (100.0)
Ampicillin, *n*=267	13 (4.9)	1 (0.6)	12 (14.3)	**˂0.001** ^**1**^	0
Ampicillin-Sulbactam, *n*=267	53 (19.9)	8 (4.6)	44 (52.4)	**˂0.001** ^**2**^	1 (12.5)
Vancomycin, *n*=267	176 (65.9)	130 (74.3)	43 (51.2)	**˂0.001** ^**2**^	3 (37.5)
Daptomycin, *n*=267	37 (13.9)	33 (18.9)	4 (4.8)	0.002^2^	0
Linezolid, *n*=267	84 (31.5)	76 (43.4)	7 (8.3)	**˂0.001** ^**2**^	1 (12.5)
Sequential antibiotic therapy, *n*=267	51 (19.1)	22 (12.6)	28 (33.3)	**˂0.001** ^**2**^	1 (12.5)
Combination therapy, *n*=267	98 (36.7)	69 (39.4)	27 (32.1)	0.256^2^	2 (25.0)
Adequate antibiotic used, *n*=267	243 (91.0)	159 (90.9)	78 (92.9)	0.589^2^	6 (75.0)
Adequate dosing and duration of anti-infective therapy, *n*=267	183 (68.5)	125 (71.4)	54 (64.3)	0.244^2^	4 (50.0)
Source control	86 (31.7)	43 (24.2)	38 (44.7)	**˂0.001** ^**2**^	5 (62.5)
Acute kidney injury	164 (60.5)	128 (71.9)	33 (38.8)	**˂0.001** ^**2**^	3 (37.5)

**Table 3:** Outcome of enterococcal bacteraemia

	Total number, *n*=271 (100%)	*E. faecium*, *n*=178 (65.7%)	*E. faecalis*, *n*=85 (31.4%)	*P* value (*E. faecium* vs. *E. faecalis*)	Other enterococci, *n*=8 (3.0%)
In-hospital mortality	89 (32.8)	72 (40.4)	16 (18.8)	**˂0.001** ^**2**^	1 (12.5)
Length of hospital stay in days, median (IQR)	38.00 (20-67)	41.50 (26-77.5)	25.00 (10-47.5)	**˂0.001** ^**3**^	34.00 (9.25-64)
ICU/IMC admission	179 (66.1)	127 (71.3)	49 (57.6)	0.027^2^	3 (37.5)
Length of ICU/IMC stay in days, median (IQR)	5.00 (0-29)	8.00 (0-35.25)	2.00 (0-16)	0.002^3^	0.00 (0-21.5)
Duration of bacteraemia in days, median (IQR) *n*=169	5.00 (2-10)	5.00 (2-10)	4.00 (1.25-11)	0.540^3^	2.00 (1,5-8)

Fisher exact test: ^1^, Chi-Square test: ^2^, Independent samples t-test: ^3^, Mann-Whitney U test: ^4^

P-values <0.001 are below the Bonferroni-adjusted level of significance of 0.000833 and are therefore considered statistically significant: **bold**

### Statistical analysis

The statistical analysis was carried out by using IBM SPSS statistic software (29.0.2.0) and R (4.3.3).

The descriptive analysis was grouped by type of *Enterococcus* (*E. faecium*, *E. faecalis*, other enterococci). Clinical differences were assessed between the two main representatives *E. faecium* and *E. faecalis* by applying statistical tests. Due to multiple comparisons, a Bonferroni correction was performed to minimize the risk of type 1 error. With a total of 60 statistical tests, the new significance level was set at 0.000833. All variables with a *p* < 0.001 fulfilled this criterion. Our initial pool of performed tests was downsized to the number of 60 because of clinical relevance and clarity.

Categorical variables were described by using absolute and relative frequencies and analysed by using the Chi-Square test or Fisher’s exact test. The Mann-Whitney U test was used for ordinal variables. Numeric variables were described by the median and interquartile range and assessed by performing the independent Samples t test.

Risk factors associated with in-hospital mortality were identified through a univariable and multivariable logistic regression analysis. Those variables that were significant at the 0.1 level in the univariable analysis were adopted in the multivariable analysis to establish the final model. Multicollinearity was assessed and ruled out. By using a conditional, forward, stepwise logistic regression approach, the final model was achieved. Goodness of fit was assessed using the Hosmer-Lemeshow test. Odds ratios are reported with corresponding 95% confidence intervals.

The performance of the DENOVA score was tested by calculating the sensitivity, specificity, positive predictive value, negative predictive value and the Youden’s index using square tables. It was completed by generating a receiver operating characteristic (ROC) curve with the DENOVA score as the test variable and IE as the state variable.

The number of available observations for each variable is reported in the tables as n = X. An acceptable maximum missingness threshold of 20% per covariate was defined.

## Results

In this study, 262 adult patients with enterococcus bacteraemia during the two-year period of 2021 and 2022 were included. Nine patients had a second case of enterococcus bacteraemia making up a total of 271 cases. The study flow chart is shown in Fig. 1. 178 cases (65.7%) were caused by *E. faecium*, 85 cases (31.4%) by *E. faecalis*, and 8 (3.0%) by other enterococci, making *E. faecium* the predominant pathogen. The other isolated pathogens were *E. avium*, *E. raffinosus*, *E. casseliflavus*, *E. hirae*, and *E. durans*. Enterococcal bacteraemia tended to be nosocomial infections (75.6%), caused by an unknown origin (39.9%), with prior use of broad-spectrum antibiotics (86.0%) within the past 6 months and mainly occurred in male patients (65.7%) with a median age of 62 years (Table 1). Regarding antibiotic resistance profiles, ampicillin- and vancomycin-resistant strains occurred significantly more often in *E. faecium* bacteraemia. 25.3% of *E. faecium* cases were VRE cases.

In contrast to a significantly higher rate of hospital-acquired infections seen in patients with *E. faecium* bacteraemia, a community-acquired acquisition was significantly more frequent in patients with *E. faecalis* bacteraemia (*p* < 0.001). Immunosuppression was found in 62% of *E. faecium* and 47% of *E. faecalis* cases.

In 61.2% of the cases, VRE screening was carried out. Patients with *E. faecium* bacteraemia were significantly more likely to have a positive VRE screening than patients with *E. faecalis* bacteraemia (34.3% vs. 7.1%, p˂0.001).

The four most common foci of infection were unknown (39.9%), abdominal (28.8%), genitourinary (15.5%) and catheter (8.1%). Whereas patients with *E. faecalis* bacteraemia were significantly more likely to have a genitourinary focus (30.6%), patients with *E. faecium* bacteraemia were significantly more likely to have an unknown (44.9%) or abdominal focus of infection (34.3%). Looking at surgery or interventions in the 6 months prior to the positive BC it was shown that more patients with *E. faecium* bacteraemia underwent cardiothoracic surgery (21.3%), whereas more patients with *E. faecalis* bacteraemia underwent urologic surgery (12.9%). Moreover, patients with *E. faecium* bacteraemia were significantly more likely to undergo an endoscopic intervention six months prior to the first positive BC (51.7%, p˂0.001) (Table 2).

Follow-up BCs were drawn in 72.1% of the cases out of which 35.6% remained positive. The median duration of bacteraemia was 5 days, with no significant difference between the different groups.

An adequate antibiotic was chosen in 91% of the cases and the dosing and duration of the therapy were adequate in 68.5% of the cases. No significant difference could be found between *E. faecium and E. faecalis* cases. Vancomycin was the most frequently used antibiotic agent (65.9%). Patients with *E. faecalis* bacteraemia were more likely to be treated with ampicillin or ampicillin-sulbactam (52.4%, p˂0.001) whereas patients with *E. faecium* bacteraemia were more likely to get treated with vancomycin (74.3%, p˂0.001) or linezolid (43.4%, p˂0.001). Sequential antibiotic therapy (33.3%, p˂0.001) and source control (44.7%, p˂0.001) occurred significantly more often in *E. faecalis* cases.

Patients with *E. faecium* bacteraemia had a significantly longer hospital stay (median: 41.5 days (26-77.5), p˂0.001) compared to patients with *E. faecalis* bacteraemia. Admission to the intensive care unit (ICU) or intermediate care unit (IMC) (71.3%, *p* = 0.027) and a longer ICU or IMC stay (median: 8 days (0-35.25), *p* = 0.002) were more often seen in patients with *E. faecium* bacteraemia, but no significant difference was shown (Table 3).

### DENOVA score

**Fig. 2:**
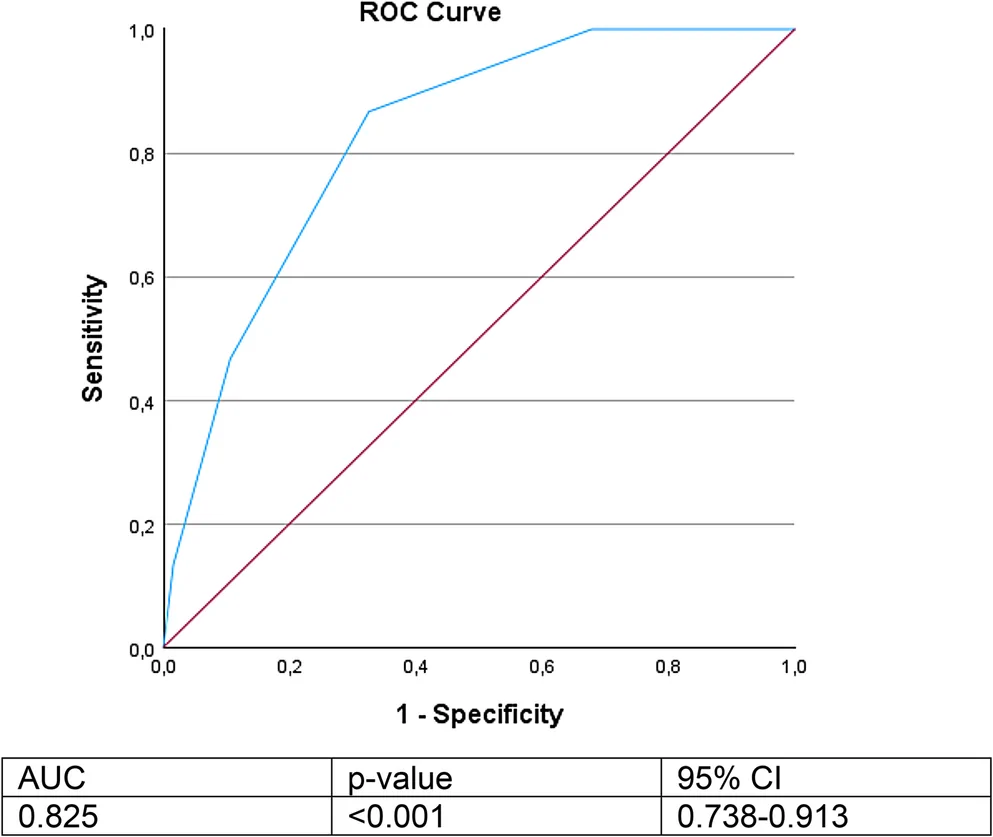
ROC curve for all cases of enterococcal bacteraemia, State variable: infective endocarditis, test variable: DENOVA score

In 35.8% of all cases, a transthoracic echocardiography was performed after the first positive BC result, the number of transoesophageal echocardiographies performed was lower (12.2%). IE was diagnosed in 5.5% of the cases. Patients with *E. faecalis* bacteraemia were more often diagnosed with IE than patients with *E. faecium* bacteraemia (10.6% vs. 3.4%, *p* = 0.024). Different cut-offs for the DENOVA score were tested for all enterococcal patients (Table 4). Choosing a cut-off of ≥ 3 led to a sensitivity of 86.67% and a specificity of 67.45%, resulting in the highest Youden Index of 0.5412, compared to a cut-off of ≥ 2 or ≥ 4. With an area under the curve (AUC) of 0.825, the model has a good discrimination ability (Fig. 2). Applying the DENOVA score with a cut-off of ≥ 3 to a subgroup of patients with *E. faecalis* bacteraemia led to a higher sensitivity, specificity and positive predictive value (Table 4). The highest Youden-Index (0.5893) could be reached by testing the DENOVA score for its originally validated cohort: monomicrobial *E. faecalis* bacteraemia.

**Table 4:** Assessment of the DENOVA score

Overview of Cut-offs for all cases of enterococcal bacteraemia (*n*=270)					
Cut-off DENOVA Score	Sensitivity	Specificity	Youden-Index	PPV	NPV
Cut-off ≥2	100% (1.0)	32.16% (0.3216)	0.3216	7.98% (0.0798)	100% (1.0)
Cut-off ≥3	86.67% (0.8667)	67.45% (0.6745)	0.5412	13.54% (0.1354)	98.85% (0.9885)
Cut-off ≥4	46.67% (0.4667)	89.41% (0.8941)	0.3608	20.59% (0.2059)	96.61% (0.9661)

Overview of diagnostic accuracy for different cohorts			
Cut-off at ≥ 3	All cases of enterococcal bacteraemia	*E. faecalis* bacteraemia	Monomicrobial *E. faecalis* bacteraemia
Sensitivity	86.67%	88.89%	87.5%
Specificity	67.45%	69.33%	71.43%
Positive Predictive Value	13.54%	25.80%	41.18%
Negative Predictive Value	98.85%	98.11%	96.15%
Youden-Index	0.5412	0.5822	0.5893

### Mortality

The overall in-hospital mortality rate amounted to 32.8%. Cases of *E. faecium* bacteraemia had a significantly higher mortality rate compared to cases of *E. faecalis* bacteraemia (40.4% vs. 18.8%, p˂0.001). In the univariable analysis source control (OR 0.605, *p* = 0.084) and bacteraemia caused by *E. faecalis* in comparison to the other pathogens (*E. faecalis*: OR 0.359, *p* = 0.001) were protective factors associated with a better survival. The Charlson Comorbidity Index, hospital-acquired infection, Pitt Bacteraemia Score, ICU/IMC admission, and polymicrobial bacteraemia were associated with a significantly higher rate of hospital mortality. The final multivariable model included the following factors independently associated with mortality: CCI (OR 1.187, *p* < 0.001), and Pitt Bacteraemia Score (OR 1.262, p˂0.001), whereas *E. faecalis* bacteraemia (OR 0.336, *p* = 0.001) was significantly associated with survival (Table 5). Multicollinearity was assessed and ruled out.

**Table 5:** Analysis of predictors of mortality using logistic regression

	Univariate Odds Ratio (OR)	*p*-value	95% CI for Odds Ratio	Multivariable Odds Ratio (OR)	*p*-value	95% CI for Odds Ratio
Age	1.008	0.372	0.991-1.025			
Female gender	0.826	0.489	0.481-1.418			
Charlson Comorbidity Index (CCI)	1.152	**0.002**	1.054-1.260	1.187	**<0.001**	1.075-1.311
Immunosuppression	1.029	0.912	0.617-1.718			
*E. faecalis* bacteraemia	0.359	**0.001**	0.193-0.666	0.336	**0.001**	0.173-0.654
Vancomycin susceptibility	0.717	0.320	0.372-1.381			
Hospital-acquired	2.143	**0.023**	1.113-4.125			
Infective endocarditis (IE)	1.024	0.967	0.339-3.090			
Pitt Bacteraemia Score	1.254	**˂0.001**	1.145-1.373	1.262	**˂0.001**	1.148-1.387
Adequate antibiotic used	1.192	0.708	0.475-2.991			
Adequate dosing and duration of anti-infective therapy	0.952	0.860	0.549-1.648			
ICU/IMC admission	3.271	**˂0.001**	1.767-6.057			
Source control	0.605	**0.084**	0.342-1.070			
Identified focus of infection	0.964	0.888	0.575-1.616			
Polymicrobial bacteraemia	1.826	**0.027**	1.071-3.116			
Combination therapy	1.163	0.576	0.686-1.971			

## Discussion

Enterococcal bacteraemia is a severe infection associated with significant morbidity and mortality, especially among elderly, immunocompromised, and multimorbid patients because of its antibiotic resistance profile and nosocomial acquisition. The in-hospital mortality of 32.8% in our cohort was among the highest of previously described mortality rates [2, 6, 8, 25]. The severity of enterococcal bacteraemia is emphasized by high ICU/IMC admission rates (66.1%), acute kidney injury (AKI) and prolonged hospital stay. Although in over 90% of the cases an adequate antibiotic was administered, the adequate dosing and duration of therapy were only chosen in 68% of the cases, thus indicating potential for optimization. Inadequate dosing and duration of therapy was often characterized by subtherapeutic dosing of Ampicillin and premature discontinuation of antibiotic therapy.

Compared to previous cohorts of enterococcal bacteraemia, it is apparent that patients in our cohort tended to be younger and more multimorbid, shown by a higher CCI [4, 6, 7, 9, 25]. Especially patients with immunosuppression (56.8%) or liver disease (29.9%) seem to be overrepresented in our cohort reflecting the fact that the MHH is a large transplant centre [6, 25]. The rate of hospital-acquired infection and the predominant occurrence in male patients were consistent with the results of earlier studies. Polymicrobial bacteraemia, antibiotic resistance, and bacteraemia caused by an unknow origin were more often represented in our cohort [6, 7]. The course of illness seemed to be more severe reflected in a higher median Pitt score [6, 26] and a higher ICU/IMC admission rate [4, 27]. These findings indicate a medically complex cohort with complicate management and limited treatment options due to unknown foci of infection and antibiotic resistance and could be explained by our specifically chosen high-risk cohort and *E. faecium* being the predominant pathogen.

The predominance of *E. faecium* bacteraemia aligns with previous results from a German study that was conducted between 2016 and 2020 [4]. An opposite allocation with predominant numbers of *E. faecalis* were found in other western countries [6, 7, 9, 18, 25, 28]. This difference might be due to the status of Hannover Medical School as a tertiary care hospital and the increasing proportion of VRE documented in several prior studies [3, 4, 8, 29]. This concerning development shows the need for early pathogen identification [10]. In our cohort 25.3% of *E. faecium* bacteraemia were caused by VRE, comparable to recent studies [3, 4, 12].

This indicates that the distinction between *E. faecium* and *E. faecalis* is crucial in everyday clinical practice. As described in previous studies, we observed that whereas patients with *E. faecalis* bacteraemia had a genitourinary focus of infection, *E. faecium* bacteraemia was more often associated with an abdominal focus of infection [6, 30]. This might be important insight for recommendations of perioperative antibiotic prophylaxis in urologic surgery. In the MHH, the recommendation for perioperative antibiotic prophylaxis for urologic procedures includes Ampicillin-Sulbactam as a single shot. No significant difference regarding immunosuppression was found. Patients with *E. faecalis* bacteraemia tended to be older, but longer hospital stays, detection of AKI and higher mortality rates were more often seen in *E. faecium* bacteraemia. This suggests that patients with *E. faecium* bacteraemia had a more severe clinical course and worse outcome. With regard to its multi-drug resistance profile and association with unknown origin, early adequate treatment with regard to known resistant profiles is key in clinical management. Indication for *E. faecium* as the causing pathogen might be prior broad-spectrum antibiotic use and hospital-acquisition [6, 30]. New additions to this list might be prior positive VRE screening and performance of endoscopy.

IE was diagnosed in 5.5% of all cases. These IE incidence numbers are lower compared to previous studies [31–34]. Limited use of echocardiographic evaluation might have led to an underdiagnosis of IE. Consistent with previous studies was the higher incidence of IE in patients with *E. faecalis* bacteraemia compared to *E. faecium* bacteraemia [6, 13]. We applied the DENOVA score with three different cut-offs in order to find out if the proposed cut-off of 3 can also be adapted to our medically complex cohort. We showed that a cut-off of 3 achieved the best balance between sensitivity and specificity with an AUC of 0.825 demonstrating a good discrimination ability. Compared to these previous studies, the sensitivity (86.67%) and the specificity (67.45%) were slightly lower [16, 17]. We also tested the DENOVA score with a cut-off of ≥ 3 for *E. faecalis* bacteraemia and monomicrobial *E. faecalis* bacteraemia. We were able to show that while the sensitivity and negative predictive value were similar for all three groups, the specificity and positive predictive value was the highest in the original validated group.

However, there are limitations of this study due to its retrospective, single-centre design. The limitations regarding generalizability were accepted in order to demonstrate characteristics of enterococcal bacteraemia at a tertiary care hospital. The data were taken from an archive system, which can result in inaccurate or missing documentation of antibiotic use, colonization, surgery or intervention in the past six months before the index BC. No imputation of missing data was performed. Missingness was assessed descriptively, and no systematic pattern of missing values was identified across the dataset. The missing data were considered to be randomly distributed. Due to its retrospective design, variables of the DENOVA score, especially subjective variables like duration of symptoms or auscultation, might have been incompletely documented leading to an underestimation of the score. In addition to that, the DENOVA score was used beyond its originally validated group despite the lower rates of IE in *E. faecium* bacteraemia. Monomicrobial *E. faecalis* bacteraemia was only detected in about 16% of the cases in our cohort and 31% in another German cohort, which creates low application possibilities for the score when only applied to the validated group [4]. It has to be acknowledged that the relatively low rate of performed echocardiographies might have led to an underdiagnosis of infective endocarditis and therefore limits the validity of the score’s performance. Because follow-up blood cultures were not drawn daily, the duration of bacteraemia might have been overestimated.

Enterococcal bacteraemia remains a significant clinical challenge with high morbidity and mortality, especially in *E. faecium* bacteraemia. Early recognition of infective endocarditis and species identification are key factors for the management of enterococcal bacteraemia. Our results imply that a broader application of the DENOVA score - outside monomicrobial *E. faecalis* bacteraemia and even in a high-risk patient cohort with an overrepresentation of immunocompromised patients like ours - might be possible and should be further evaluated in prospective studies with larger cohorts.

## Supplementary Information

Below is the link to the electronic supplementary material.

Supplementary file1 (DOCX 14 KB)

## Data Availability

No datasets were generated or analysed during the current study.
