# Supplementary material for: Clinical characteristics, risk factors, and outcome of Enterococcal bacteraemia: Assessment of the DENOVA score in a diverse patient cohort
Source: Infection. 2026 Jun 17;54(5):2409–18. doi: 10.1007/s15010-026-02856-1 (PMC13727848; doi:10.1007/s15010-026-02856-1)
Supplement: Supplementary file 1 [file 15010_2026_2856_MOESM1_ESM.docx]

**6) Supplementary material:**

|  | Total number, n=271 (100%) | *E. faecium*, n=178 (65.7%) | *E. faecalis*, n=85 (31.4%) | P value (*E. faecium* vs. *E. faecalis*) | Other enterococci n=8 (3.0%) |
| --- | --- | --- | --- | --- | --- |
| Clinical Frailty Scale (CFS), median (IQR), n=229 | 4.00  (3-5) | 4.00  (3-5) | 4.00  (3-6) | 0.014^4^ | 3.00  (2-5) |
| Community acquired | 66 (24.4) | 14 (7.9) | 48 (56.5) |  | 4 (50.0) |
| **Focus of infection** |  |  |  |  |  |
| Spondylodiscitis | 4 (1.5) | 0 | 4 (4.7) |  | 0 |
| Osteomyelitis | 2 (0.7) | 0 | 2 (2.4) |  | 0 |
| Soft tissue | 11 (4.1) | 6 (3.4) | 5 (5.9) |  | 0 |
| Other foreign bodies | 4 (1.5) | 1 (0.6) | 3 (3.5) |  | 0 |
| Time to Positivity (TTP) (hours), median (IQR) | 12.07  (8,75-14,58) | 13.165 (9.9325-15.55) | 10.37  (6.97-13.05) | 0.612^3^ | 12.75 (7.985-4.43) |
| Prior enterococcal colonization in the past 6 months | 147 (54.2) | 102 (57.3) | 43 (50.6) | 0.306^2^ | 2 (25.0) |
| **Catheter present at index culture** |  |  |  |  |  |
| Peripheral venous catheter | 103 (38.0) | 48 (27.0) | 51 (60.0) | **˂0.001^2^** | 4 (50.0) |
| Central catheter | 206 (76.0) | 154 (86.5) | 47 (55.3) | **˂0.001^2^** | 5 (62.5) |
| Arterial catheter | 101 (37.3) | 75 (42.1) | 25 (29.4) | 0.047^2^ | 1 (12.5) |
| ECMO | 17 (6.3) | 15 (8.4) | 2 (2.4) | 0.061^2^ | 0 |
| Total number of different catheters, median (IQR) | 2.00 (1-3) | 2.00 (1-4) | 2.00 (1-3) | 0.009^3^ | 1.50 (1-2) |
| **DENOVA score** |  |  |  |  |  |
| Duration of symptoms ≥ 7 days, n=270 | 120 (44.4) | 75 (42.1) | 41 (48.8) | 0.310^2^ | 4 (50.0) |
| Embolization | 65 (24.0) | 48 (27.0) | 15 (17.6) | 0.098^2^ | 2 (25.0) |
| Valve disease | 130 (48.0) | 81 (45.5) | 47 (55.3) | 0.137^2^ | 2 (25.0) |
| Auscultation of heart murmur | 13 (4.8) | 12 (6.7) | 1 (1.2) | 0.005^1^ | 0 |
| Total number of positive blood cultures, median (IQR) | 1.00 (1-2) | 1.00 (1-3) | 2.00 (1-2) | 0.493^3^ | 1.00 (1-1) |
